# Ginger Ingredients Alleviate Diabetic Prostatic Complications: Effect on Oxidative Stress and Fibrosis

**DOI:** 10.1155/2017/6090269

**Published:** 2017-08-17

**Authors:** Basma G. Eid, Hala Mosli, Atif Hasan, Hany M. El-Bassossy

**Affiliations:** ^1^Department of Pharmacology and Toxicology, Faculty of Pharmacy, King Abdulaziz University, Jeddah 21589, Saudi Arabia; ^2^Department of Medicine, Faculty of Medicine, King Abdulaziz University, Jeddah 21589, Saudi Arabia; ^3^Department of Anatomy and Embryology, Faculty of Veterinary Medicine, Kafrelsheikh University, Kafrelsheikh 33516, Egypt

## Abstract

Prostatic complications are common in patients with diabetes. This study investigated the effect of different ginger ingredients: zingerone, geraniol, and 6-gingerol on the prostate in diabetic rats. Diabetes was induced in Wistar rats by streptozotocin intraperitoneal injection (50 mg/kg), and the rats were left for 10 weeks to develop prostatic complications. In diabetic treated groups, rats received daily oral zingerone, geraniol, and 6-gingerol in doses of 20, 200, and 75 mg/kg, respectively, in the last 8 weeks. Treatment with the compounds caused changes in the ventral prostate of diabetic animals as indicated by the columnar ductal epithelium and dense secretions. There was an amelioration of oxidative stress as evidenced by the lowering of prostate malondialdehyde and elevating prostate oxidized to reduced glutathione (GSH/GSSG) ratios by geraniol and 6-gingerol. None of the three ginger ingredients affected the hyperglycemia, reduction in body weight gain, and testosterone deficiency seen in diabetic animals. Interleukin-1*β* and interleukin-6 levels remained unchanged. However, zingerone and geraniol ameliorated the fibrosis in diabetic prostate through suppressing the elevated prostate transforming growth factor beta 1 (TGF*β*1) and collagen IV. Therefore, ginger ingredients could be beneficial in alleviating diabetic prostatic complications through suppressing oxidative stress and tissue fibrosis.

## 1. Introduction

Prostatic complications are commonly reported in patients with diabetes mellitus [1]. The pathology leading to these complications is complex and requires further exploration. It has been suggested that steroidal signaling and lipid and peptide signaling play a major role in developing prostatic complications [1–4]. Insulin resistance is a clinical state in which the response to insulin is compromised and below normal, frequently leading to hyperinsulinemia. This insulin resistance has been linked to an increased risk of prostatic complications [5].

Oxidative stress is the excessive release of reactive oxygen species (ROS). When associated with hyperglycemia, oxidative stress has been postulated to be one of the hallmarks of the development of complications associated with diabetes, mainly via the polyol pathway [6, 7]. Oxidative stress has detrimental consequences on the male genital system by compromising the sperm cells and spermatogenesis as well as causing testicular damage [8]. Microvascular complications as a result of oxidative stress have been reported to occur with diabetes mellitus [9]. As a result, we postulated that the prostatic damage that may occur due to hyperglycemia may be eminent from these microvascular changes that occur due to the excessive release of ROS during oxidative stress leading to inflammation of the prostate.

Ginger* (Zingiber officinale)* is a plant which is popular in homeopathy and alternative medicine and is used to treat a number of diseases [10]. Gingerols and zingerone have been identified as two important active constituents of ginger that have various biological effects [11]. In particular, 6-gingerol was recognized to have antihyperglycemic, anti-inflammatory, antiangiogenic, and cytotoxic effects on cancer cells [12–15]. Ginger has also been found to possess androgenic activity in male rats [16]. Since 6-gingerol and zingerone are active constituents present in ginger and ginger was found to have anti-inflammatory as well as androgenic activity, this suggests that these compounds may have a protective effect on the prostate gland. Ginger was recently reported to have positive effects on sperm mobility and viability and could be beneficial to maintain healthy male reproductive function [17]. Geraniol is a monoterpene compound that is also present in ginger. It has been shown to possess anti-inflammatory as well as antioxidant effects [18–20].

These studies suggest that microvascular changes may occur in the prostate gland due to oxidative stress as a result of hyperglycemia. They also suggest that the components of ginger, namely, 6-gingerol, zingerone, and geraniol, could provide a protective effect on the prostate gland by acting as antioxidants and improving the overall male reproductive state. The aims of the current study were to (a) evaluate the protective effect of 6-gingerol, zingerone, and geraniol on the histological changes that occur in the prostate gland; (b) to evaluate the protective effect of 6-gingerol, zingerone, and geraniol on serum levels of various markers of oxidative stress as well as some inflammatory cytokines; and (c) to evaluate the protective effect of 6-gingerol, zingerone, and geraniol on key markers of fibrosis using immunofluorescence.

## 2. Materials and Methods

The research methodologies are in accordance with the Regulations of Research Bioethics on the Living Creatures of the National Committee of Bio. & Med. Ethics, Kingdom of Saudi Arabia.

### 2.1. Study Protocol

#### 2.1.1. Experimental Design

Male Wistar rats (7 weeks old; King Abdulaziz University, KSA) were randomly divided into five groups (8 animals each); the control (C) group received the vehicle for a total duration of 10 weeks. Streptozotocin (50 mg/kg) was injected IP to induce diabetes. The diabetic (D) group received streptozotocin and the vehicle. The diabetic-6-gingerol (DG) group received 6-gingerol (75 mg/kg/day) orally by gavage two weeks after streptozotocin injection for 8 weeks. The diabetic-zingerone (DZ) group received zingerone (20 mg/kg/day) orally by gavage two weeks after streptozotocin injection for 8 weeks. The diabetic-geraniol (DR) group received geraniol (200 mg/kg/day) orally by gavage two weeks after streptozotocin injection for 8 weeks.

A glucose meter was used to check tail blood glucose levels (ACCU-CHEK; Hoffman-La Roche Ltd., Basel, Switzerland). A blood sugar level of 250–350 mg dL^−1^ two weeks after STZ injection confirmed diabetes and diabetic complications developed after an additional 8 weeks [21]. Animals were sacrificed by decapitation and using guillotine. The ventral prostate of all the rats was harvested. A portion of the prostate tissue was kept in liquid nitrogen and later stored at −75°C. The other portion was fixed in 0.1 M phosphate-buffered 10% formalin (pH 7.4) overnight and embedded in paraffin for histopathology. 5-*μ*m thick sections from each sample were prepared and mounted on poly-L-lysine coated glass slides.

### 2.2. Studying the Effect of Zingerone and Geraniol and 6-Gingerol, on Prostatic Changes That Occur in Streptozotocin-Induced Diabetes Mellitus

At the end of study, the effects of 6-gingerol, zingerone, and geraniol administration on prostatic changes due to diabetes mellitus were determined through the following.

#### 2.2.1. Histopathological Studies

Samples of the ventral prostate were collected from all animal groups (6 rats/group) and fixed in a 10% buffered formalin solution overnight. The samples were then processed and embedded in paraffin. 5 *μ*m thick sections were obtained by rotatory microtome; then sections were stained with Harris' hematoxylin & eosin and Masson's trichrome stains, according to standard procedures. Stained sections were examined by a light microscope to detect histopathological changes.

Epithelial heights were obtained by using image analyzer software (Leica Qwin 500). Twenty readings from random fields of each stained section (5 rats/group) were recorded at high magnification (×400).

#### 2.2.2. Biochemical and ELISA Measurements

Tissue extraction reagent I of the following composition: 50 mM Tris, pH 7.4; 250 mM NaCl; 5 mM EDTA; 2 mM Na_3_VO_4_; 1 mM NaF; 20 mM Na_4_P_2_O_7_ and 0.02% NaN_3_ (Invitrogen Corporation, Camarillo, CA, USA) was used to homogenize the snap frozen tissues under cooling. Centrifugation at 10,000 rpm was carried out for 5 minutes with cooling. An analysis of the supernatant was carried out to determine the level of malondialdehyde and glutathione-glutathione reductase ratio in the prostate homogenate using commercially available kits (Abcam®, Cambridge, MA, USA). Prostatic IL-6 and IL-1*β* and serum testosterone were measured by ELISA kit (Abcam, Cambridge, MA, USA) using primary antibodies raised against rat IL-6 and IL-1*β* and testosterone, respectively.

#### 2.2.3. Immunofluorescence Studies

Five *μ*m sections (6 sections from 6 different animals per group) of the ventral lobe of the prostate were stained using TGF*β*1 and collagen IV antibodies for immunofluorescence studies. The slides were heated using a hot plate and subsequently deparaffinized using xylene and placed in ethanol and distilled water. The slides were then placed in hydrogen peroxide kept at −20°C for half an hour. Antigen retrieval was carried out using a citrate buffer at 95°C for half an hour and then washed using PBS. The slides were then kept in a humidified chamber. Nonspecific binding sites were blocked using bovine serum albumin in PBS with 5% normal goat serum, 1% BSA, and 0.1% Triton for half an hour at ambient temperature. Subsequently the sections were kept in PBS for 35 min for washing. Incubation overnight with the primary antibody diluted in the blocking buffer at 4°C was next performed. The sections were then washed three times (5 min) using PBS and then incubated with a fluorescent conjugated secondary antibody that was diluted 1 : 200 in blocking buffer for 1 hour in the dark. The sections were then washed three times (5 min) using PBS, dried, and mounted with “Prolong” mounting media (Life Technologies, Paisley, UK). The slides were kept in the dark until the next day before they were studied using Zeiss LSM 780 immunofluorescence microscope (Carl Zeiss, Gottingen, Germany) at wavelengths (488 and 561 nm excitation and 497–542 emission filters). Minimal excitation and gain using the same parameters were used to obtain the images. For quantification, one image was used for each animal. Image fluorescence intensity was measured by Zen software (Carl Zeiss, Gottingen, Germany) on the captured unmanipulated images. Representative of the captured whole unmanipulated fluorescence images were presented in results figures. No fluorescence was detected in the sections treated only with the secondary antibody. The primary chicken polyclonal antitransforming growth factor beta 1 (TGF*β*1) and the mouse monoclonal anti-collagen I antibody (both at 1 : 1000, Abcam, Cambridge, MA, USA) and the secondary Alexa Fluor (*λ*ex = 555 and *λ*ex = 488, resp.) conjugated goat anti-chicken and goat anti-mouse (both at 1 : 200, Life Technologies, Grand Island, NY, USA) were used.

### 2.3. Statistical Analysis

Data were analyzed by ANOVA and Newman-Keuls' post hoc test. They are shown as mean ± SE of the mean. *p* < 0.05 indicated statistical significance. Statistical analyses were conducted using Prism 5 (GraphPad, CA, USA).

## 3. Results

### 3.1. Zingerone, Geraniol, and 6-Gingerol Improved the Histopathological Profile of Streptozotocin-Induced Diabetic Rats

Significant changes in glandular epithelial heights were recorded (Table 1). In diabetic group (D), there was sharp decrease in epithelial height when compared with that of the control group (C). On the other hand, in diabetic treated rats, there was significant increase in epithelial height with geraniol (DR) and 6-gingerol (DG) diabetic treated groups; the highest value was recorded with zingerone (DZ) diabetic treated group (Table 1); in spite of this, epithelial height in all diabetic treated rats did not reach that of the control rats (Table 1).

**Figure 1:**
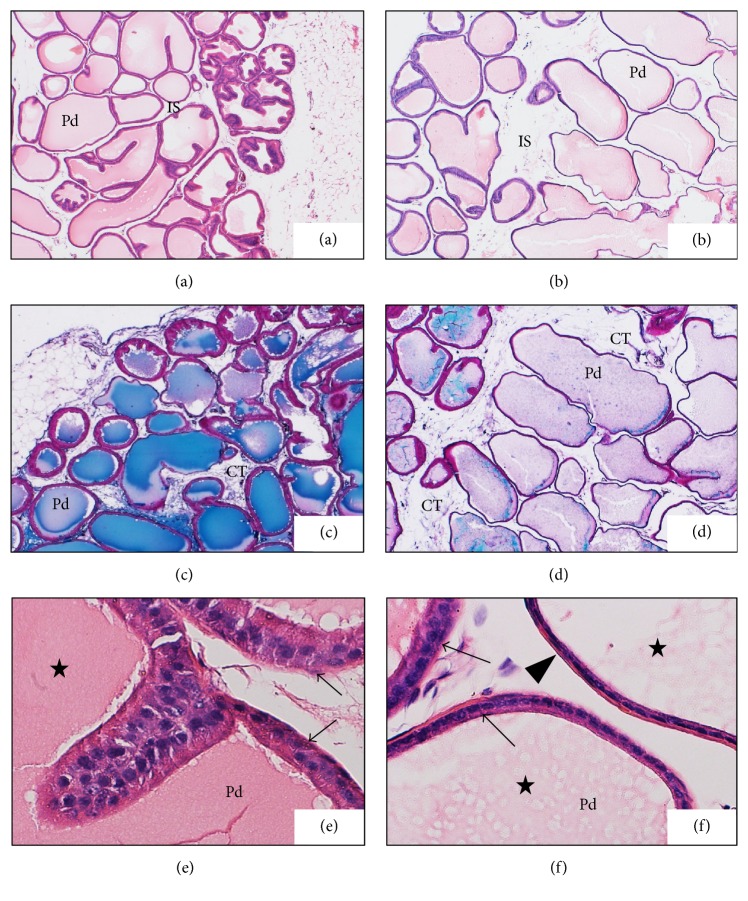
Photomicrographs show comparison between ventral prostate of control (a, c, e) and diabetic rats (b, d, f). Prostatic ductules (Pd) in control rats (a) were adhered together with minimal interductal spaces (IS) and had thick walls but in diabetic rats (b) their walls were relatively thin with marked irregular outlines (H&E ×40). Interductal spaces (IS) were wider in diabetic rats (d) and filled with connective tissue (CT) while, in control rats (c), connective tissue (CT) was a little amount between ductules (Masson's trichrome ×40). Most of epithelial cells in control rats (e) were columnar cells (arrows) while in diabetic rats (f) they were cuboidal (arrow) and even flat cells (arrow head). Secretions (stars) were darkly stained in control rats (e) and lightly stained in diabetic rats (f) (H&E ×400).

**Figure 2:**
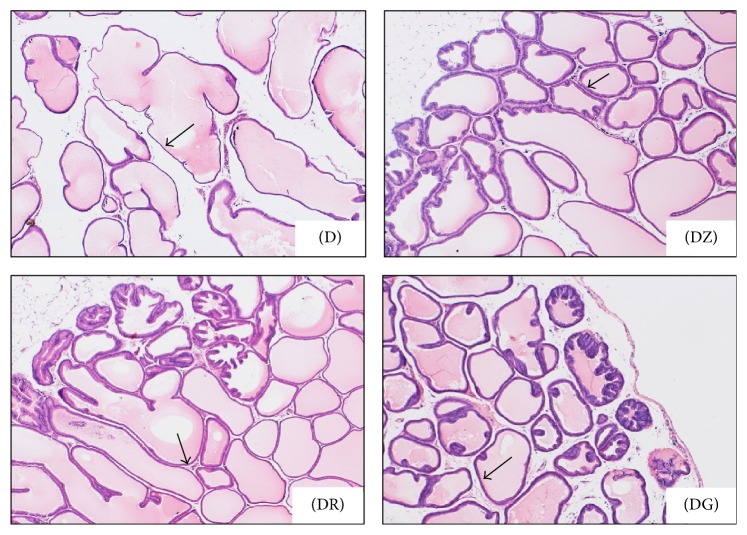
Ventral prostate of diabetic rats (D) and zingerone treated (DZ), geraniol treated (DR), and 6-gingerol treated (DG) diabetic rats. Wide interductal spaces were prominent in diabetic rats (D) in comparison with the diabetic treated groups (DZ, DR, and DG). In diabetic rats, ductal walls (arrows) were thin and undulated, while, in treated groups, ductal walls were relatively thicker and more uniform. Epithelial folds were always present in the external glandular layer in diabetic treated groups (H&E ×40).

Epithelial folding in diabetic and diabetic treated groups was not a prominent feature. Although epithelial folds were always present in the superficial layers of prostatic ductules in all examined groups (Figures 1(a), 2(DZ), 2(DR), and 2(DG)), it was minimal in the diabetic group (Figure 1(d))

Changes in the ductal epithelium of the rat ventral prostate in control rats, diabetic rats (D), zingerone treated rats (DZ), geraniol treated rats (DR), and 6-gingerol treated rats (DG) were presented in H&E stained sections (Figures 1(e), 1(f), and 4). The ductal epithelium was low (low cuboidal to flat) in diabetic rats when compared with control rats (Figures 1(e) and 1(f)). In diabetic treated rats, the ductal epithelium was mostly columnar being taller than that of diabetic rats; the tallest epithelial cells were present in zingerone treated rats (DZ) (Figure 4).

**Figure 3:**
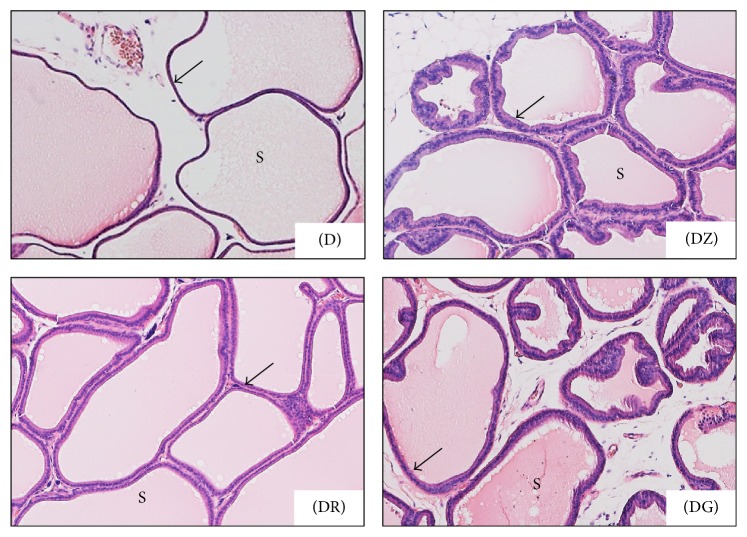
Higher magnification of the preceding photomicrograph, ventral prostate in different groups; diabetic rats (D) and diabetic treated rats (DZ; DR; DG). Prostatic secretions (S) were darkly stained in diabetic treated groups when compared with diabetic ones; ductal walls (arrows) were clearly thicker in treated groups while, in diabetic group, they were thin (H&E ×100).

**Figure 4:**
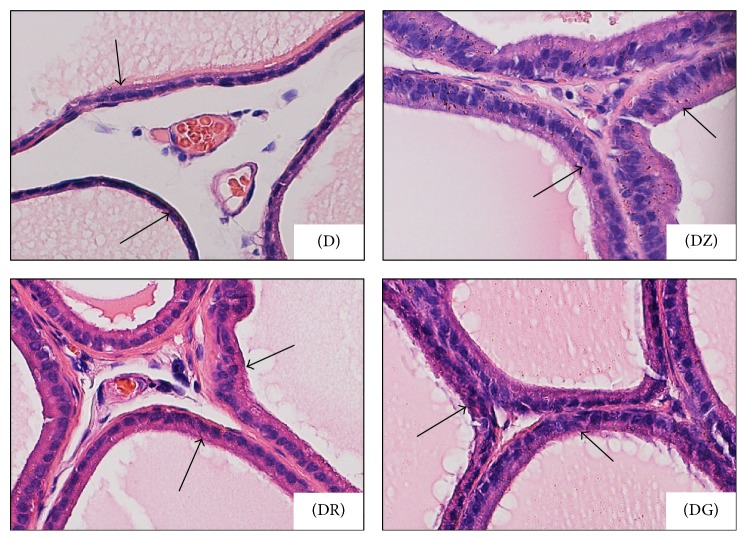
Photomicrographs show changes in the ductal epithelium (arrows) of the rat ventral prostate; diabetic rats (D), zingerone treated rats (DZ), geraniol treated rats (DR), and 6-gingerol treated rats (DG). Epithelial height was markedly increased in diabetic treated rats ((DR) and (DG)) and reached its highest limits in DZ group (H&E ×400).

In diabetic rats (D), walls of prostatic ducts were thin and undulated (Figures 1(b), 1(d), 2(D), and 3(D)); relatively thicker ductal walls were seen in diabetic treated groups (Figures 3(DZ), 3(DR), 3(DG), 4(DZ), 4(DR), and 4(DG)). Regarding prostatic secretions, in all groups, the secretions were acidophilic (stained pink with H&E and blue with Masson's trichrome). In the diabetic group (D), they were scanty lightly stained (Figures 1(b), 1(d), 1(f), 3(D), and 4(D)) while, in the other groups, they stained darker (Figures 1(a), 1(c), 1(e), 3(DZ), 3(DR), 3(DG), 4(DZ), 4(DR), and 4(DG)).

**Figure 5:**
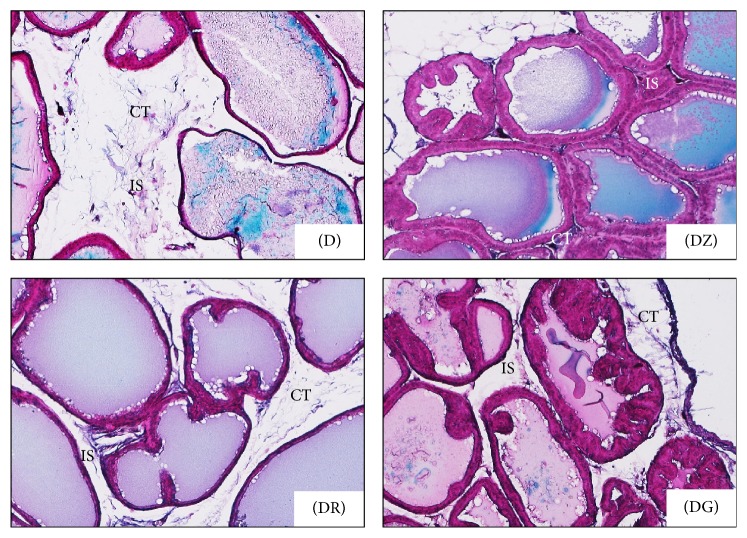
Photomicrographs of rat ventral prostate show the interductal spaces (IS) in different groups; diabetic rats (D) and diabetic treated rats ((DZ), (DR), and (DG)). Relatively wide interductal spaces were recorded in diabetic group (D) and it contained connective tissue (CT) elements (Masson's trichrome ×100).

In the diabetic group (D), wider interductal spaces (IS) appeared between prostatic ducts (Figures 1(b) and 1(d)) when compared with that of the control group (Figures 1(a) and 1(c)). In diabetic treated groups, the interductal spaces (IS) became narrower (Figures 3(DZ), 3(DR), and 3(DG)) when compared with that of diabetic group (Figure 3(D)). The relatively wider interductal spaces in diabetic group (D) were filled with connective tissue elements mostly collagenous fibers (Figure 5(D)), and fewer connective tissue elements were found in the diabetic treated groups (Figures 5(DZ), 5(DR), and 5(DG)).

### 3.2. Effects of Zingerone, Geraniol, and 6-Gingerol on Postprandial Glucose Levels, Body Weight Gain Percent, and Testosterone Levels

**Figure 6:**
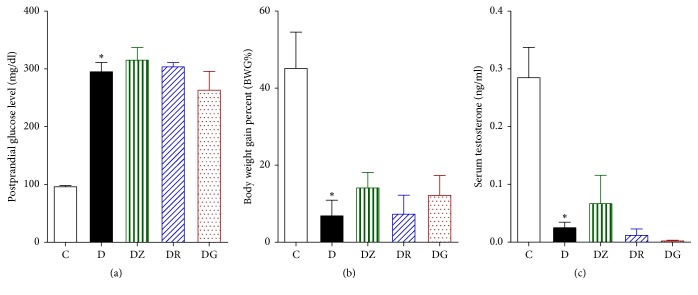
Measurements of postprandial glucose levels (mg/dl) (a), body weight gain percent (BWG%) (b), and serum testosterone levels (ng/ml) (c) in control (C), diabetic rats (D), zingerone treated rats (DZ), geraniol treated rats (DR), and 6-gingerol treated rats (DG), respectively (data are expressed as mean ± SE of the mean; ^*∗*^*p* < 0.05 in comparison to controls (C)).

Postprandial glucose levels were found to be threefold higher in diabetic rats than in control rats (Figure 6(a)). These levels remained high after treatment with zingerone, geraniol, and 6-gingerol, with no significant difference noted between the treated groups and the diabetic group. Body weight gain percentage (BWG%) was found to be significantly lower in diabetic rats in comparison to control rats (Figure 6(b)). No significant difference was noted between the treated groups and the diabetic group. Serum testosterone levels were found to be significantly lower in diabetic rats in comparison to control rats (Figure 6(c)), with no significant differences found between the diabetic rats and the treated rats.

### 3.3. Geraniol and 6-Gingerol Ameliorated Key Oxidative Stress Markers

**Figure 7:**
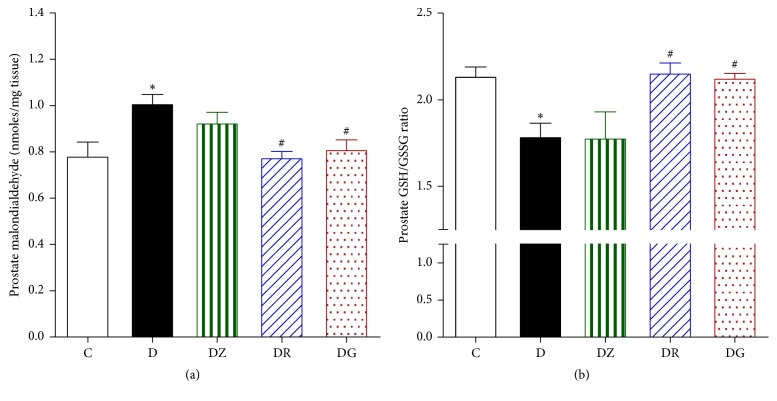
Measurement of the prostate malondialdehyde (a) and prostate GSH/GSSG ratios (b) in homogenized prostate tissues in control (C), diabetic rats (D), zingerone treated rats (DZ), geraniol treated rats (DR), and 6-gingerol treated rats (DG), respectively (data are expressed as mean ± SE of the mean; ^*∗*^*p* < 0.05 in comparison to controls (C); ^#^*p* < 0.05 in comparison to diabetic rats (D)).

**Figure 8:**
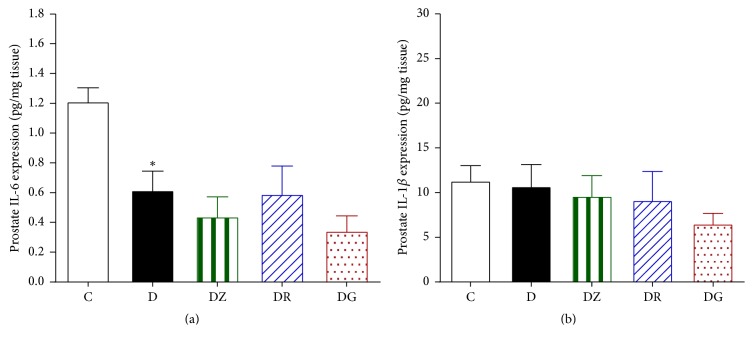
Measurements of the levels of IL-6 (a) and IL-1b (b) expression in the prostate in control (C), diabetic rats (D), zingerone treated rats (DZ), geraniol treated rats (DR), and 6-gingerol treated rats (DG), respectively (data are expressed as mean ± SE of the mean; ^*∗*^*p* < 0.05 in comparison to controls (C)).

The levels of prostate malondialdehyde were found to be higher in diabetic rats in comparison to control rats (Figure 7(a)). Treating the rats with zingerone did not ameliorate these levels; however treatment with geraniol and 6-gingerol was found to decrease the levels of malondialdehyde significantly in comparison to the diabetic levels (Figure 7(a)). The levels of prostate GSH/GSSG ratio were found to be lower in diabetic rats compared to control rats (*p* < 0.05) (Figure 7(b)). Treating the rats with geraniol and 6-gingerol returned the prostate GSH/GSSG ratio to control levels (*p* < 0.05). Zingerone treatment, however, had no significant effect on the prostate GSH/GSSG ratio in comparison to the diabetic animals (Figure 7(b)). The levels of IL-6 were found to be lower in diabetic rats in comparison to control rats (*p* < 0.05), and these levels were not affected by treatment (Figure 8(a)). The levels of prostate IL-1b expression were similar in control, diabetic, and drug-treated animals (Figure 8(b)).

### 3.4. Zingerone and Geraniol Produce a Decrease in the Expression of TGF*β*1 and Collagen IV

**Figure 9:**
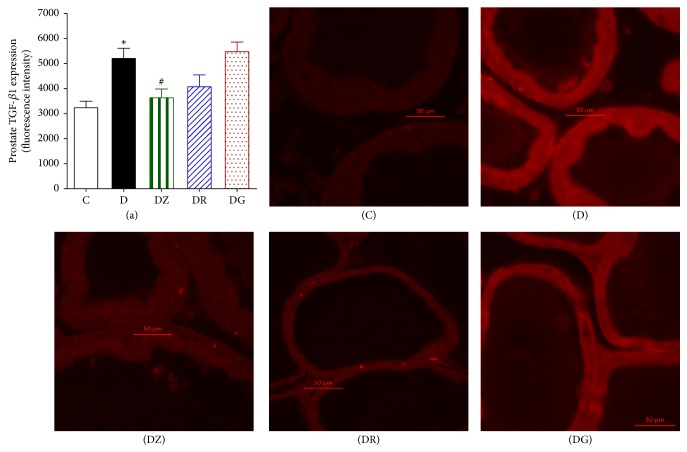
Prostate TGF*β*1 immunofluorescence in streptozotocin-induced diabetic rats, control (C), diabetic rats (D), zingerone treated rats (DZ), geraniol treated rats (DR), and 6-gingerol treated rats (DG). The primary chicken polyclonal antitransforming growth factor beta 1 (TGF*β*1) and the mouse monoclonal anti-collagen I antibody and the secondary Alexa Fluor (*λ*ex = 555 and *λ*ex = 488, resp.) conjugated goat anti-chicken and goat anti-mouse were used (data are expressed as mean ± SE of the mean; ^*∗*^*p* < 0.05 in comparison to controls (C); ^#^*p* < 0.05 in comparison to diabetic rats (D)).

**Figure 10:**
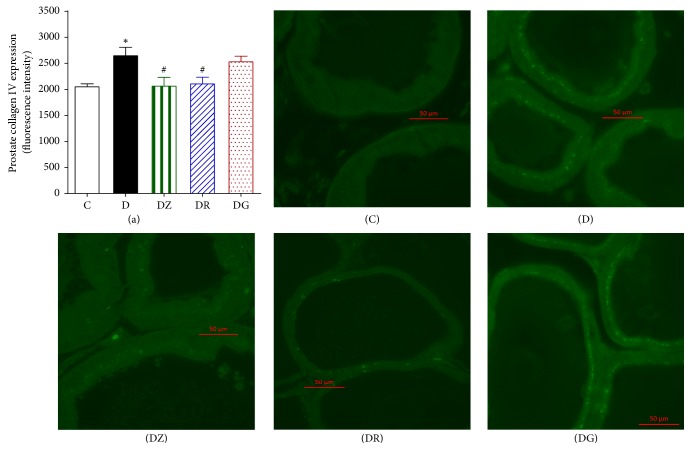
Prostate collagen IV immunofluorescence in streptozotocin-induced diabetic rats, control (C), diabetic rats (D), zingerone treated rats (DZ), geraniol treated rats (DR), and 6-gingerol treated rats (DG). The primary chicken polyclonal antitransforming growth factor beta 1 (TGF*β*1) and the mouse monoclonal anti-collagen I antibody and the secondary Alexa Fluor (*λ*ex = 555 and *λ*ex = 488, resp.) conjugated goat anti-chicken and goat anti-mouse were used (data are expressed as mean ± SE of the mean; ^*∗*^*p* < 0.05 in comparison to controls (C); ^#^*p* < 0.05 in comparison to diabetic rats (D)).

Sections of the prostate displayed a clear increase in the immunofluorescence for TGF*β*1 (*p* < 0.05) when compared to the control group (Figures 9(a) and 6(b)). Zingerone treatment decreased this elevated fluorescence, whereas geraniol and 6-gingerol had no effects. Similarly there was a notable increase in the fluorescence of prostate collagen IV in diabetic rats in comparison to the control group (*p* < 0.05) (Figures 10(a) and 7(b)). Treatment with zingerone and geraniol caused a marked decrease in the fluorescence of collagen IV (*p* < 0.05) whereas 6-gingerol treatment had no effect.

## 4. Discussion

The aim of the current study was to investigate the prostatic changes that occur in a streptozotocin-induced diabetic rat model, as well as the protective effect of 6-gingerol, zingerone, and geraniol on these changes. This was carried out by isolating the ventral lobe of the prostate gland from each group of rats and examining the histological changes that occur, in addition to immunofluorescence studies.

Upon histopathological examination of stained sections of the ventral prostate, signs of necrosis and leukocytic infiltration were absent. The gland of diabetic rats looked less active in comparison with the normal prostate in control rats. Ductules were enlarged with irregular outlines and thin walls. The epithelial lining was low in height ranging from being cuboidal to being flat. Epithelial folds were absent and lumina were filled with scanty lightly stained acidophilic secretions. Wide interductal spaces between ductules were a prominent feature; these spaces were filled with connective tissue.

Diabetic rats treated with the tested substances showed improvement of the profile picture of the ventral prostate. This improvement was apparent in the ductules, where the lining epithelium ranged from cuboidal to columnar cells. The tallest columnar epithelial cells were present in prostate of zingerone treated rats. Secretions were dense darkly stained acidophilic fluids (pink with H&E and blue with MT). The interductal spaces were narrow and connective tissue elements between ductules were sparse if compared with that of diabetic rats. These changes reflect an amelioration of the histopathological changes that occurred in diabetic rats.

It is known that the rat ventral prostate is a compound ductal gland and its ductules are lined mainly by columnar epithelium [22]. Induction of diabetes mellitus by streptozotocin caused marked adverse histological changes in rat ventral prostate; the epithelial lining became low, ranging from low cuboidal till flattened cells, and the ductules walls became thin and undulated; similar results were obtained in ventral prostate of diabetic rats [23, 24] and a marked decrease in ventral prostate weight was also recorded. In mice, streptozotocin-induced diabetes caused also reduction in epithelial height [25]; on the other hand, alloxan induced diabetes caused intense epithelial atrophy beside signs of prostatic inflammation and malignancy [26]; these latter complications were not observed in our study. It is known that the normal prostatic secretion is slightly alkaline, because of this it attains the color of the acidic dyes (pink in H&E and blue in Masson's trichrome), so that it is acidophilic, and it is also milky in consistency. In case of diabetic rats, some findings as low epithelial height, thin walled ductules, and lightly colored and scanty secretions indicate that this gland is less active when compared with that of control rats; similar findings were recorded by Abd El-Haleem and Zidan (2011).

Previous studies stated that diabetes caused adverse effects in different body systems including urogenital system [27] and impair the testicular function [28]; a recent study recorded that induced diabetes had adverse effects on ventral prostate of rat during sexual maturation [23]; another study suggested that depletion of testosterone and insulin resulted in severe atrophy in rat prostate [29]. Although, in the present study, testosterone levels were significantly lower in diabetic rats than in control rats, these levels were not changed by treatment of diabetic rats with zingerone, geraniol, and 6-gingerol suggesting that these compounds were having a direct effect on the prostate rather than acting through raising testosterone levels.

A constant finding in streptozotocin-induced diabetic rats was the decreased weight of ventral prostate [30–32]; this was histologically evident through the low ductal epithelium and the scanty secretions; it seems that the prostate gland of the STZ-induced diabetic rats was affected through the lack of the anabolic activity of insulin [23, 30, 31] and it was deprived of the regulatory mechanisms of testosterone which plays a basic role in the prostate gland development, epithelial proliferation, and normal secretory function [33]; on the other hand, a previous study confirmed that STZ-induced diabetes decreased the serum level of testosterone in rats [30]; a recent study mentioned that both insulin and testosterone are essential for cell proliferation in rat prostate [32].

In the current study, connective tissue elements specially collagen increased in the wide interductal spaces in diabetic rats; it was detected by histological and immunofluorescence techniques; similar results were recorded in the same species [24, 34]; it is said that the increased collagen fibers and stromal cells in the prostate of diabetic rat would create a reactive stromal microenvironment that predisposes to prostatic hyperplasia [34–36].

Malondialdehyde levels serve as a measure of ROS, which can have detrimental effects on male reproductive organs [37, 38]. The levels of prostate malondialdehyde were measured as an indicator of lipid peroxidation and were found to be higher in diabetic rats when compared to control rats. Geraniol and 6-gingerol reduced the levels of malondialdehyde thus exhibiting a protective effect on the prostate. This is in accordance with previous studies, where* Z. officinale *reduced malondialdehyde levels and showed a protective antioxidant effect by a reduction in the level of malondialdehyde in male rats [39] and improved the oxidative stress levels.

The prostate GSH/GSSG ratio was significantly lower in diabetic rats in comparison to control rats. Administration of geraniol and 6-gingerol improved the prostate GSH/GSSG ratio to levels comparable with controls. These results are similar to previously published work in which ginger was found to have powerful antioxidant properties due to the action of its active ingredients including zingerone, gingerols, and shogaols [17, 40, 41].

IL-6 has been reported as a major mediator of acute inflammatory reactions [42]. Previous studies have shown elevated levels of IL-6, which would be expected in the presence of inflammation [43]. Our study found a reduction in the levels of IL-6 in diabetic rats in comparison to control rats and similar IL-1b expression in control, diabetic, and treated groups. It is possible that other cytokines were involved in this inflammatory process. Levels of IL-6, IL-8, TNF-*α*, and IGF-1 and clusterin levels were all reported by other studies to be elevated in prostatic inflammation [43].

TGF*β*1 is a major cytokine in inflammation that is as a key player in the regulation of stromal differentiation and proliferation in benign prostatic hyperplasia. The expression of TGF*β* receptor II protein expression in patients suffering from BPH was studied and a correlation between TGFBRII stromal staining and the volume of the prostate was reported [44]. In our study there was a significant increase in the immunofluorescence for TGF*β*1 which was reduced by zingerone. In addition, we observed a significant increase in the fluorescence of prostate collagen IV in diabetic rats in comparison to controls. This level was lowered by zingerone and geraniol treatment indicating reduced fibrosis.

In men who suffer from diabetes mellitus and prostate complications, the polyuria caused by hyperglycemia in combination with lower urinary tract symptoms could be problematic and this combination may potentially interfere with activities of daily living leading to social isolation and dependency. This may feasibly lead to further deterioration of the patient's health as well as psychological state. While treatments for the underlying problems do exist, many patients prefer to minimize the number of medications they are taking while leaning towards therapies derived from nature. Our study demonstrated that the products studied while overall safe had the added benefit of exhibiting a protective effect on prostate tissue exposed to toxic environment in the form of hyperglycemia-induced inflammation. We demonstrated that the tested products ameliorated the tissue damage caused by the inflammatory milieu in addition to effectively lowering ROS products. Further human research is needed to confirm that these findings translate into clinical outcome.

## 5. Conclusion

This study has provided direct evidence of a link between diabetes mellitus and prostatic complications and demonstrated that the three active ginger compounds, namely, zingerone, geraniol, and 6-gingerol, provide a protective effect on the prostate as evidenced by an improvement of the histopathological profile as well as a reduction in inflammatory processes.

## Figures and Tables

**Table 1:** Mean values of glandular epithelial height of rat ventral prostate.

Animal groups	C	D	DZ	DR	DG
Epithelial height (*μ*m)	14.34 ± 2.48	4.27 ± 1.50^*∗*^	12.83 ± 2.21^#^	7.34 ± 0.98^#^	6.96 ± 1.33^#^

Data are expressed as mean ± SD; SD = standard deviation; C: control rats, D: diabetic rats, DZ: zingerone treated diabetic rats, DR: geraniol treated diabetic rats, and DG: 6-gingerol treated diabetic rats (^*∗*^*p* < 0.05 in comparison to controls (C); ^#^*p* < 0.05 in comparison to diabetic rats (D)).
